# A Cracking Model for Reinforced Concrete Cover, Taking Account of the Accumulation of Corrosion Products in the ITZ Layer, and Including Computational and Experimental Verification

**DOI:** 10.3390/ma13235375

**Published:** 2020-11-26

**Authors:** Tomasz Krykowski, Tomasz Jaśniok, Faustyn Recha, Michał Karolak

**Affiliations:** 1 Department of Mechanics and Bridges, Faculty of Civil Engineering, Silesian University of Technology, Akademicka 5, 44-100 Gliwice, Poland; faustyn.recha@polsl.pl; 2 Department of Building Structures, Faculty of Civil Engineering, Silesian University of Technology, Akademicka 5, 44-100 Gliwice, Poland; tomasz.jasniok@polsl.pl; 3 3D Team Oddział Wrocław, Wrocławski Park Biznesu, Bierutowska 57-59, 51-317 Wrocław, Poland; m.karolak@3dteam.pl

**Keywords:** reinforcement corrosion, cover cracking, accelerated corrosion test, optical measurements, FEM, elastic-plastic-fracturing model, corrosion strain tensor

## Abstract

The paper presents the finite element method model (FEM) which allows the forecasting of the evolution of damage in a concrete cover together with experimental verification of the model. The objective of the model is to define the corrosive volume strain tensor rate effected by corrosion, which comprises the accumulation of corrosion products in pore spaces as well as in micro-cracks which develop at the initial stage of cover degradation. The propagation of damage in the contact zone was captured by taking into account the function describing the degradation of the interface transition zone depending on the cover tightening time–critical time. The method of determining the critical time along with the method of taking into account the effective electrochemical equivalent of iron was also analyzed in this paper. The work presents the experimental verification of the model using an accelerated corrosion test of reinforcement in concrete and strain measurements with optical methods. The conducted tests demonstrate satisfactory compliance of the model with the test results.

## 1. Introduction

The damage to reinforced concrete elements caused by reinforcement corrosion is a very important problem pertaining to the operation of reinforced concrete structures. The reasons for electrochemical corrosion of reinforcing steel used in reinforced concrete elements stem principally from the carbonization process of concrete cover resulting in the decrease in concrete pH value or/and the diffusion process of chloride ions to the surface of reinforcing bars or direct current leaks from electric tractions [1]. The regulations used in the design of structural elements enforce the obligation to use covers of appropriate thickness [2,3], but in reality this thickness may be insufficient and can initiate corrosive processes resulting in a progressive decrease in the load capacity of reinforced concrete elements. Good knowledge of corrosion process mechanisms allows the determination of both the effects of corrosion (e.g., cross-sectional reduction of rebars or the place of developed cracks), and the estimation of the time at which such effects can occur [4].

**Figure 1:**
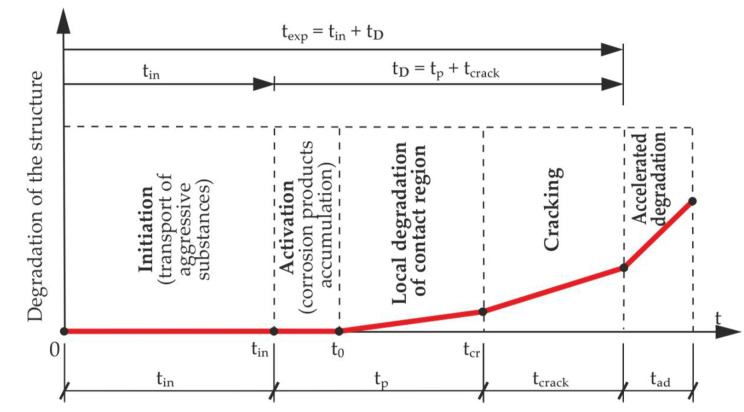
Scheme of degradation of the structure over time.

Reinforcement corrosion processes are complex problems, and two major phases related to the life time of the structure $t_{\exp}$ can be basically distinguished in them. These are the phases of electrochemical corrosion initiation (in) and the degradation phase (D), which comprises the work of the corrosion cell and the propagation of mechanical damage in the cover. Schematically the process of degradation can be simplified according to the scheme shown in Figure 1. This figure describes the characteristic stages of the corrosion process causing degradation of reinforced concrete elements under the conditions of reinforcement corrosion given in the literature e.g. [4], modified here in order to include the very important changes in the contact zone of steel and concrete): where: $t_{\mathrm{in}}$ is the reinforcement corrosion initiation time, $t_{p}$ is the propagation time, $t_{0}$ is the time of activation of mechanical impact by corrosion products, $t_{\mathrm{cr}}$ is the critical time, $t_{\mathrm{crack}}$ is the cracking time, $t_{\mathrm{ad}}$ is the accelerated degradation of the structure time and $t_{D}$ is the time of degradation.

The initiation phase of the reinforcement corrosion process has been described in detail in the literature [5,6,7]. It is assumed that exceeding the critical concentration of chloride ions or the critical pH value in concrete leads to the development of an electrochemical cell [8,9] and consequently, to the flow of the corrosion current and to the development of corrosion products that tightly fill up the free pore spaces around the reinforcing bar in the so-called transition layer [10]. Another effect that has an impact on the evolution of damage in the cover is the propagation of micro-cracks in the structure of the contact layer steel-concrete [11,12,13].

The literature offers a lot of models which enable the description of the incremental process of volumetric strains effected by corrosion products [14,15]. The vast majority of these models ignore the influence of the porosity of the transition layer and the propagation of micro-cracks in the cover, or they significantly simplify this process [16]. The problem covering the accumulation of corrosion products in the transition layer has been analyzed, among others, in the paper [17], where the propagation of corrosion products in the pore structure surrounding the rebar was analyzed. A different approach is proposed in [12,13], where an a priori concept of the volume of corrosion accommodation region (car) is proposed. The model for the corrosion degradation of the cover, presented in these papers, erroneously assumes that the corrosion process of the reinforcement proceeds in a uniform manner around the circumference of the rebar. In the general situation this assumption is incorrect and is also not consistent with the method for carrying out the research formulated in [13]. A significant problem in the approach formulated in [13] is also the lack of a clear method for adopting the key parameters of the model calculations, such as the cumulative volume of the corrosion products. This undoubtedly influences the method of describing the effect of corrosion products on the structure of the cover. An additional problem is the assumption adopted in many papers, which says that the entire mass of corrosion products produced spreads in the plane perpendicular to the rebar axis, which, according to the authors of this paper, is not true at the initial stage of the process.

The present work clarifies the theoretical concept involving the description of the developmental process of corrosive damages in the cover, using the tensor of corrosive volumetric strain rate [18]. Moreover, this research analyzes the model, along with its numerical verification, which enables the recognition of the deposition of corrosion products in the pores of the transition layer and in micro-cracks developed in the initial phase of the corrosion process. The paper formulates the equations for the evolution of the corrosive volumetric strain rates as a function of the parameter β (this parameter depends on the activation time $t_{0}$ and the critical time $t_{\mathrm{cr}}$ and determines the progress of cover degradation). The paper presents a method of experimental estimation of the critical time $t_{\mathrm{cr}}$, which is a key parameter for model description at the initial stage of cover degradation. The method of analysis of the reinforcement corrosion processes proposed in the paper allows the inclusion of the problems of non-regular distribution of corrosion products on the circumference of the rebar as well as for the changes in the nature of the volumetric strain velocity field. The presented theoretical considerations have been supported by experimental research aimed at determining many key parameters of the model, critical time $t_{\mathrm{cr}}$ and the effective electrochemical equivalent of reinforcing steel $k_{\mathrm{eff}}$. The work also includes an evaluation of compliance of the obtained results of experimental research with theoretical calculations. The experimental research on reinforcement corrosion processes was carried out using the so-called accelerated corrosion test [19] and optical strain measurement techniques [20].

## 2. Calculation Model

**Figure 2:**
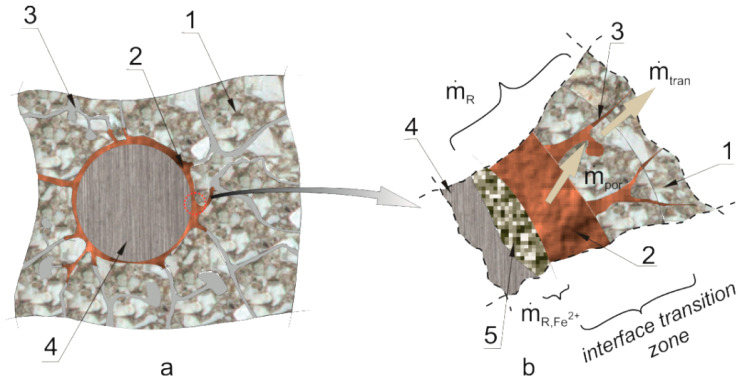
The model of corrosion product deposition in concrete pores: (**a**) interface steel-concrete transition zone, (**b**) corrosion product mass flows in the interface transition zone steel-concrete – description in the text.

In reinforced concrete elements in which the reinforcement corrosion process is initiated, mechanical effects are not initiated at the same time when the reinforcement corrosion process is activated. This effect is fundamentally connected with the microstructure of the transition layer [16,17] and with the propagation of micro-damages in the initiation phase of the reinforcement corrosion process [13]. The mechanisms of this process are presented in a simplified manner in Figure 2, where Arabic numerals denote: (1) cement grout, (2) products from reinforcement corrosion, (3) pore voids (with air and pore solution), (4) steel rebar, (5) corrosion pit filled with products from reinforcement corrosion.

The effective mass balance of the corrosion products that mechanically impacts on the cover structure of the rebar can be expressed with the following relationship:

(1) $${\dot{m}}_{\mathrm{eff}}={\dot{m}}_{\mathrm{ekw}}-{\dot{m}}_{\mathrm{por}}-{\dot{m}}_{\mathrm{tran}},\;{\dot{m}}_{\mathrm{ekw}}={\dot{m}}_{R}-{\dot{m}}_{R,{\mathrm{Fe}}^{2+}},\;$$

where ${\dot{m}}_{\mathrm{eff}}$ is the effective mass change rate of corrosion products, ${\dot{m}}_{R}$ is the source of the mass of corrosion products—the accumulation rate of corrosion products in the investigated area, ${\dot{m}}_{\mathrm{por}}$ is the mass change rate of corrosion products resulting from their being carried away to the micro-cracks and micro-slits formed in the interface transition layer as a result of mechanical impact, ${\dot{m}}_{\mathrm{tran}}$ is the rate of mass change of corrosion products due to the transport process of the products into deeper concrete layers, ${\dot{m}}_{R,{\mathrm{Fe}}^{2+}}$ is the rate of mass change of corrosion products due to their deposition in the corrosion pitting of the rebar, and this is proportional to the rate of the development of this loss, in line with Faraday’s law

(2) $${\dot{m}}_{{\mathrm{Fe}}^{2+}}=k_{\mathrm{comp}}I_{\mathrm{corr}},{\;k}_{\mathrm{comp}}=k_{\mathrm{comp}}\left(k\right),$$

where $I_{\mathrm{corr}}$ is the total intensity of corrosive current flowing in the microcells, $k_{\mathrm{comp}}$ is the computational electrochemical equivalent which comprises all deviations from ideal conditions, and which will be developed in Section 3 of the work, k is the electrochemical equivalent of iron determined in line with the equation

(3) $$k=\frac{M}{\mathrm{zF}}$$

In Equation (3) M is the molar mass of iron equal to 56 g/mol, F is the Faraday constant equal to $F=9.6485\times 10^{4}\;C/\mathrm{mol}$, and z is the reaction charge number equal to 2.

Assuming a small thickness of the interface transition layer, and assuming a constant density of the corrosion products $\rho_{R}$, the Equation (1) can be formulated in the form [17,18,21]

(4) $${\dot{V}}_{\mathrm{eff}}={\dot{V}}_{\mathrm{ekw}}-{\dot{V}}_{\mathrm{por}}-{\dot{V}}_{\mathrm{tran}},\;{\dot{V}}_{\mathrm{ekw}}={\dot{V}}_{R}-{\dot{V}}_{{\mathrm{Fe}}^{2+}}$$

Where ${\dot{V}}_{\mathrm{eff}}$ is the rate of change of effective volume of corrosion products with a mass of $m_{\mathrm{eff}}$, ${\dot{V}}_{R}$ is the rate of change in mass of products from reinforcement corrosion with a mass of $m_{R},$ ${\dot{V}}_{{\mathrm{Fe}}^{2+}}$ is the rate of change in volume of corrosion loss with a mass of $m_{{\mathrm{Fe}}^{2+}}$ tightly filled with corrosion products with volume $V_{{\mathrm{Fe}}^{2+\;}}=V_{R,{\mathrm{Fe}}^{2+\;}}$ and mass $m_{R,{\mathrm{Fe}}^{2+}\;}$, ${\dot{V}}_{\mathrm{por}}$ is the rate of change in volume of corrosion products transferred into micro-cracks formed in the interface transition layer, while ${\dot{V}}_{\mathrm{tran}}$ is the rate of change in the volume of corrosion products transferred outside the interface transition layer deeper into the concrete cover. The determination of volume change rates ${\dot{V}}_{\mathrm{por}}$ and ${\dot{V}}_{\mathrm{tran}}$ is very complex from the theoretical point of view, so it was assumed that these quantities change in a manner proportional to the rate of the change of the equivalent volume ${\dot{V}}_{\mathrm{ekw}}$ [18]:

(5) $${\dot{V}}_{\mathrm{eff}}=\left(1-\beta \right){\dot{V}}_{\mathrm{ekw}},\;{\dot{V}}_{\mathrm{ekw}}=\psi I,{\;\beta \;\dot{V}}_{\mathrm{ekw}}={\dot{V}}_{\mathrm{por}}+{\dot{V}}_{\mathrm{tran}}$$

(6) $$\psi =\frac{\left(\alpha^{-1}\vartheta -1\right)k}{\varrho_{{\mathrm{Fe}}^{2+}}},\;\alpha =m_{{\mathrm{Fe}}^{2+}}m_{R}^{-1},\vartheta =\varrho_{{\mathrm{Fe}}^{2+}}\varrho_{R}^{-1}$$

where ψ is a constant depending on the composition of corrosion products, $\varrho_{{\mathrm{Fe}}^{2+}}$ is the density of the mass of iron ions, $\varrho_{R}$ is the density of corrosion products, α and ϑ are parameters adopted depending on the chemical composition of corrosion products [14].

The effect of change over time involving the impact of corrosion products on the concrete of the cover was defined using the parameter β. It was assumed in the research that β can be any function, but to simplify the considerations it was assumed that the said relation is linear [13] (the linear form of the β function was shown in this paper for the first time) [18]:

(7) $$\beta =\{\begin{array}{cc} 1, & t<t_{0}\;\left(V_{\mathrm{ekw}}<V_{\mathrm{ekw}}^{0}\left(t_{0}\right)=V_{\mathrm{por}}\right)\\ (t_{\mathrm{cr}}-t)/\left(t_{\mathrm{cr}}-t_{0}\right), & t_{0}\leq t\leq t_{\mathrm{cr}}\;\left(V_{\mathrm{ekw}}^{0}\left(t_{0}\right)\leq \;V_{\mathrm{ekw}}\left(t\right)\leq V_{\mathrm{ekw}}\left(t_{\mathrm{cr}}\right)\right)\\ 0, & t>t_{\mathrm{cr}}\;\left({\;V}_{\mathrm{ekw}}\left(t\right)>V_{\mathrm{ekw}}\left(t_{\mathrm{cr}}\right)\right). \end{array}$$

**Figure 3:**
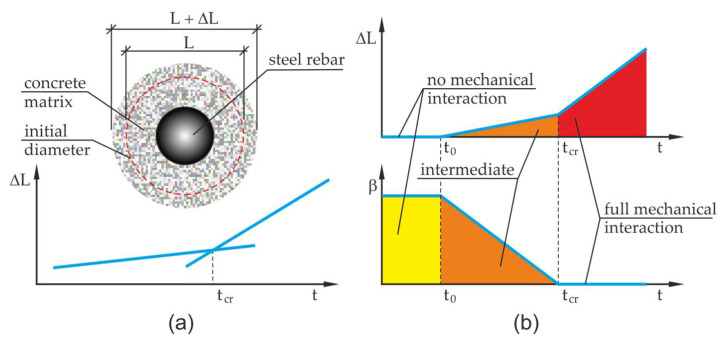
Methodology for determining critical time $t_{\mathrm{cr}}$ and function β: (**a**) method of determination of critical time as the intersection point of regression lines, (**b**) course of changes of function β together with the description of the impact of corrosion products on concrete.

The function β described by the Equation (7) comprises three main phases that occur during the course of reinforcement corrosion processes. The times $t_{0}$ and $t_{\mathrm{cr}}$ define the characteristic work phases of the corrosion cell. Time $t_{0}$ is the time needed for corrosion products to fill up free pore spaces located in the transition layer $V_{\mathrm{ekw}}<V_{\mathrm{ekw}}^{0}\left(t_{0}\right)=V_{\mathrm{por}}$. The next stage is the time interval from $t_{0}\;$to the critical time $t_{\mathrm{cr}}$. This stage of the corrosion process is connected with a gradual increase of mechanical impacts which result from the increase of pressure exerted by corrosion products on the concrete of the cover. At that stage, apart from the compaction of the layer of corrosion products, there is also a propagation of micro-cracks taking place in the cover and in the transition layer, into which the corrosion products penetrate, gradually blocking the possibility of carrying away the corrosion products into deeper layers of the cover. The critical time described in the literature involving such problems can be identified with the concept of volume and the evolution of cumulative accommodation region [13]. Yet, the application of this concept is rather complicated since it is difficult to experimentally assess this volume of the developed corrosion products and assuming a predetermined value of the critical time is a burden of error. By using the notion of critical time $t_{\mathrm{cr}}$ and measurements of displacements in the test this quantity can be relatively easily approximately determined in an experimental way, (compare Figure 3), where L is the basis for measurement of the test element, ΔL is the elongation of this length.

This operation can be made by determining the intersection point of two approximated straight lines characterizing the increments of displacement in the analyzed sample before and after exceeding the critical time (it is necessary to continuously measure the test element to have the necessary displacement data to find approximated straight lines). It is assumed in the analysis that the corrosion products are not pushed away deep into the developing crack at the initial stage of the corrosion process). By allowing for the definition of the time derivative of the first invariant of volumetric corrosion strain tensor

(8) $${\dot{I}}_{1\varepsilon^{V}}≅\frac{{\dot{V}}_{\mathrm{eff}}}{V_{0}}≅{\dot{\varepsilon }}_{11}^{V}+{\dot{\varepsilon }}_{22}^{V}+{\dot{\varepsilon }}_{33}^{V}≅{\eta \dot{\varepsilon }}^{V}$$

where ${\dot{\varepsilon }}_{\mathrm{ii}}^{V}$ are the components of volumetric strain tensor rate in the tangent direction to the reinforcement axis ${\dot{\varepsilon }}_{33}^{V}$ and in the plane perpendicular to the reinforcement axis respectively ${\dot{\varepsilon }}_{11}^{V}$ and ${\dot{\varepsilon }}_{22}^{V}$.

Depending on the phase of the analyzed corrosion process, three situations can occur. The first is for $t<t_{0},$ the parameter η in Equation (8) takes the value η=3, tensor of volumetric strain rates is an isotropic tensor with non-zero coordinates equal to ${\dot{\varepsilon }}_{11}^{V}={\dot{\varepsilon }}_{22}^{V}={\dot{\varepsilon }}_{33}^{V}={\dot{\varepsilon }}^{V}.$ The next situation takes place for time $t>t_{\mathrm{cr}}$ the coordinate of tensor of volumetric strain rate ${\dot{\varepsilon }}_{33}^{V}$ in the direction tangent to the axis of the reinforcing bar ${\dot{\varepsilon }}_{33}^{V}=0$, the parameter η takes the value η=2, non-zero coordinates of tensor of volumetric strain rates in the perpendicular plane to the bar axis are equal to ${\dot{\varepsilon }}_{11}^{V}={\dot{\varepsilon }}_{22}^{V}={\dot{\varepsilon }}^{V}$. The last considered situation is the intermediate situation, which takes place in the time interval $t_{0}\leq t\leq t_{\mathrm{cr}},\;$the parameter η takes variable values from the interval η∈(2,3), η=3β+2(1−β), the coordinate of tensor of volumetric strain rate ${\dot{\varepsilon }}_{33}^{V}$ in the direction tangent to the bar axis is described by the function ${\dot{\varepsilon }}_{33}^{V}={\dot{\varepsilon }}_{33}^{V}\left(\beta \right),$ ${\dot{\varepsilon }}_{33}^{V}\in \left(0,{\dot{\varepsilon }}^{V}={\dot{\varepsilon }}_{11}^{V}={\dot{\varepsilon }}_{22}^{V}\;\right)$, while the non-zero coordinates of the tensor of volumetric strain rates in the plane perpendicular to the bar axis take the values ${\dot{\varepsilon }}_{11}^{V}={\dot{\varepsilon }}_{22}^{V}={\dot{\varepsilon }}^{V}$.

The state of strain depends here on the impact stage of the corrosion process on the concrete of the cover. With the rise of pressure, which goes along with the filling up process of free pore spaces by corrosion products, the character of the strain state changes to a plain strain state. The phases of the reinforcement corrosion process and their corresponding forms of volumetric strain tensor corrosion rates were determined by the Equations (9) and (10)

- Spatial state of strain, $t\leq t_{\mathrm{cr},}$
  (9) $${\dot{\varepsilon }}_{\alpha \beta }^{V}={\dot{\varepsilon }}^{V}\delta_{\alpha \beta }=\frac{\left(1-\beta \right)\left(\alpha^{-1}\vartheta -1\right)k_{\mathrm{comp}}I_{\mathrm{corr}}}{{\eta \rho }_{{\mathrm{Fe}}^{2+}\;}V_{0}}\delta_{\alpha \beta },\;\eta =3\beta +2\left(1-\beta \right),{\dot{\varepsilon }}_{33}^{V}={\beta \;\dot{\varepsilon }}^{V}.$$
- Flat state of strain, $t>t_{\mathrm{cr},}$
  (10) $${\dot{\varepsilon }}_{\alpha \beta }^{V}={\dot{\varepsilon }}^{V}\delta_{\alpha \beta }=\frac{\left(\alpha^{-1}\vartheta -1\right)k_{\mathrm{comp}}I_{\mathrm{corr}}}{{\eta \rho }_{{\mathrm{Fe}}^{2+}\;}V_{0}}\delta_{\alpha \beta },{\dot{\varepsilon }}_{33}^{V}=0,\;\eta =2,$$
  where $V_{0}$ is initial volume of the region of the body being subject to volumetric strains and α, β are the indexes, α, β=1,2.

## 3. Experimental Research

**Figure 4:**
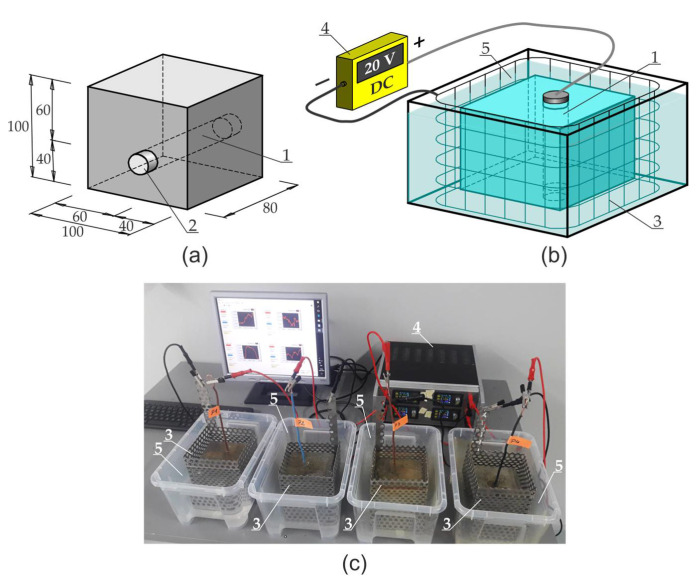
Stand for testing accelerated corrosion of the reinforcement using the potentiostatic method: (**a**) schematic of the sample with the location of reinforcing bar, (**b**) measuring system, (**c**) view of the stand with power supply unit and four samples–description in the text.

In order to assess the compliance of the proposed theoretical model with the experimental tests, an accelerated corrosion test was carried out on reinforcing bars 20 mm in diameter, made from St3SX steel (Poland – designation from the production date of the bar and the chemical composition of steel was given in the Table 1) and placed in four C50/60 concrete samples P1, P2, P3, P4 measuring 100 × 100 × 80 mm. The concrete mix was made off the river aggregate of CEM I cement (Górażdże Cement SA, Chorula, Poland – chemical composition of cement is presented in Table 2) without admixtures and additions at w/c = 0.4. After concreting, samples were demolded after 24 h and then stored in a chamber (Feutron GmbH, Langenwetzendorf, Germany) at 20 °C and 90% humidity for 3 months. The axonometry of the sample and the location of the rebar 1 is presented in Figure 4a. The front surfaces of the bar 1 were protected with polyester resin (Fenedur S.A., Montevideo, Uruguay) 2 before the development of corrosion processes in that area of the bar.

**Figure 5:**
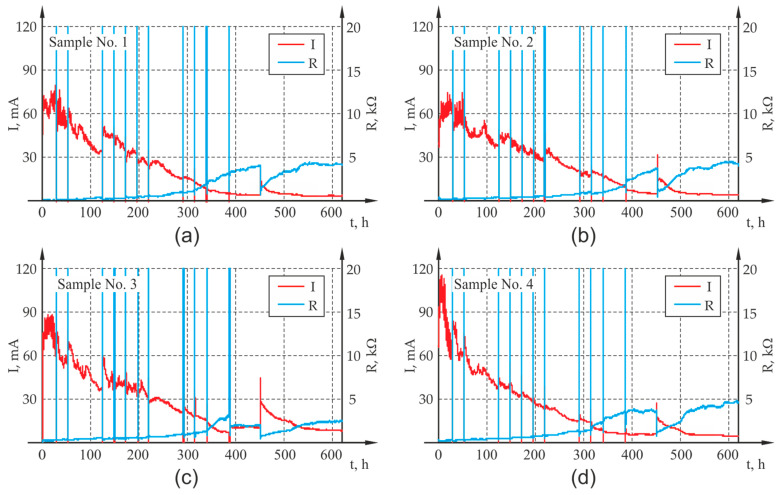
Results of the measurement of current I and electrical resistance of the system R carried out during the test: (**a**) P1, (**b**) P2, (**c**) P3, (**d**) P4.

The accelerated corrosion test was carried out with the use of an electrolyser (Silesian University of Technology, Gliwice, Poland) (Figure 4b), which forced corrosion of the reinforcement uniformly around the circumference of the rebar. The reinforcing bar 1 was the anode, while the cathode was made of weather-resistant perforated steel sheet 3, which surrounded the concrete sample on all sides. Both electrodes were connected to power supply 4 with insulated wires. The sample was immersed in tap water 5 ensuring electrical contact of all electrodes of the system. The applied power supply unit 4 enabled a simultaneous connection and independent measurement of four samples (Figure 4c). The generated voltage for all channels was equal and had the value of 20 V throughout the entire test period. The power supply unit enabled automatic recording of basic electrical parameters of the system, i.e., current I, voltage U and electrical resistance of the system R with the preset frequency of 60 s. The results of the measurement were presented as graphs illustrating the course of changes in current and electrical resistance in the analyzed samples, which for all four samples are presented in Figure 5 out during the test: (a) sample P1, (b) sample P2, (c) sample P3, (d) sample P4.

The use of direct current significantly accelerates the course of the experiment, ensuring homogeneity of corrosion and enables the collection of precise data necessary for modelling the phenomenon. On the other hand, the acceleration of the process causes that the decomposition of corrosion products in concrete is different than in the case of natural corrosion, which is of key importance when comparing the results to corroded real structures. In addition, monitoring the rate of naturally occurring corrosion by electrochemical methods is very difficult and can often be burdened with a large error.

The applied voltage brought about a significant shift of the potential of the steel electrode, where iron digestion was the dominant process

(11) $$\mathrm{Fe}-2e¯\to {\mathrm{Fe}}^{2+}$$

Taking into account the above dominant process, the mass of oxidizing iron ions was calculated from Faraday’s law,

(12) $${\bar{m}}_{{\mathrm{Fe}}^{2+}}=\overset{t}{\underset{0}{\int }}{\mathrm{kI}}_{\mathrm{ext}}\mathrm{dt}$$

whereas compared to the formula (2), ${\bar{m}}_{{\mathrm{Fe}}^{2+}}$ is the mass of iron ions carried away from the steel rebar to the concrete microstructure after the duration time t of the electrolysis, k is the electrochemical equivalent determined in compliance with the Equation (3), while the current intensity is not related to the flow of charge in the corrosion microcells, but it is the total intensity of the external current flowing in the electric circuit $I_{\mathrm{ext}}$.

The indiscriminate calculation of the coefficient k from the formula (3) equal to $k=\;2.894\times 10^{-4}\;g/C\;=0.00912\;g/\mu \mathrm{AYear}\;$and including it in formula (12) may lead to large discrepancies between the actual mass of iron ions transferred to concrete microstructure measured gravimetrically and that calculated from the formula (12). Numerous research studies with the application of an electrolyser used to accelerate the corrosion process of reinforcement in concrete have unequivocally proved that there are differences between the gravimetrically measured loss of steel ${\bar{m}}_{g}\;$and that calculated from the Faraday’s law ${\bar{m}}_{{\mathrm{Fe}}^{2+}}$ based on Equation (12). The acquisition of consistent results requires the adoption in the tests of the effective electrochemical equivalent of the process of accelerated corrosion of the reinforcement $k_{\mathrm{eff}}=k_{\mathrm{eff}}(\lambda_{g-F})$ where $\lambda_{g-F}$ is the relative difference in mass determined by the formula

(13) $$\lambda_{g-F}=\frac{{\bar{m}}_{{\mathrm{Fe}}^{2+}\;}-{\bar{m}}_{g}}{{\bar{m}}_{{\mathrm{Fe}}^{2+}\;}},{\;k}_{\mathrm{eff}}=\left(1-\lambda_{g-F}\right)k$$

When analyzing the literature in the field of accelerated corrosion tests on reinforcement in concrete, a large discrepancy in the obtained results can be observed. The discrepancies of λ_g-F_, e.g., in the paper [22], did not exceed 5%, while in [23] the relative difference was larger and ranged from 5% to 25% depending on the applied intensity of the electric current. Much higher values of λ_g-F_, from 2% even up to 200%, were obtained in [24], but here the discrepancies involved both the overestimation and underestimation of the mass of iron ions in relation to gravimetric measurements. The studies presented in [25,26] an electrolyzer were also used to accelerate the corrosion of reinforcement, but the ranges of the abovementioned relative differences were not defined. Yet, it was pointed out that the reaction (11) is not the only one that takes place at the anode, and the calibration of results is needed after the termination of the experiment. The above conclusions and research results prompted us to conduct our own research, in which the impact of cement type (cements CEM I, CEM II (Górażdże Cement SA, Chorula, Poland) and CEM III (Górażdże Cement SA, Chorula, Poland) were taken into account), and the impact of the method of concrete curing on the λ_g-F_ values were estimated. The research demonstrated that the greatest relative differences of up to 65% were obtained for the cement CEM I, and the smallest about 21% for the cement CEM III. And good curing of concrete, i.e., storing the samples in limewater until the tests were carried out, resulted in the rise of the λ_g-F_ value to about 63% for CEM I, 45% for CEM II and 34% for CEM III. On the other hand, the lack of curing (without the samples being in contact with water after demolding) caused the relative mass differences to be much smaller, 35%, 27% and 21%, respectively. Therefore, it can be assumed that the better the curing conditions the tighter the microstructure of the concrete, and the λ_g-F_ values are higher.

In the above research, a test was also carried out on bars in concrete of the same specifications and using the same curing method as for the samples P1, P2,P3, and P4 marked respectively as P5, P6, and P7. Before concreting, the bars were weighed, and after the completion of the tests, the samples were split and the bars were taken out, and after cleaning them from corrosion products, they were reweighed. The mean value of λ_g-F_ determined with the Equation (13) was here about 40%. The results of the calculations carried out along with the determination of the resulting values of the effective electrochemical equivalent of iron $k_{\mathrm{eff}}$ and the average value $k_{\mathrm{eff},\mathrm{avg}}$ of this parameter used in further calculations are summarized in Table 3.

**Figure 6:**
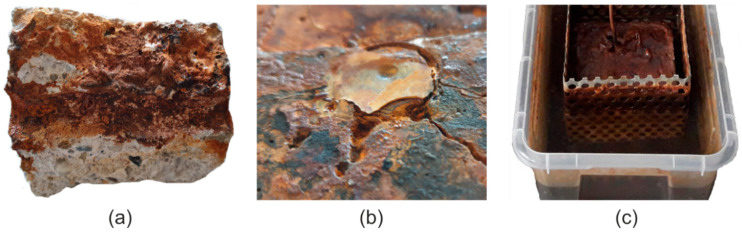
Accumulation of corrosion products of the rebar steel during experimental tests: (**a**) sample break in the fracture, (**b**) detachment of resin from the bar face, (**c**) contaminated water and container with corrosion products.

The experimental tests revealed that from the image of crack surface after splitting (Figure 6a), from a visual inspection of the rebar’s face in the area of the applied resin (Figure 6b) and from the contamination of water and the container with corrosion products (Figure 6c) that some part of the corrosion product is washed out into the solution and does not accumulate at the rebar surface as assumed by the calculation model. Failure to estimate this aspect would lead to further errors in the calculation model. To determine the mass of corrosion products that entered the solution, after the completion of the tests the entire liquid was filtered through a medium filter and the mass of the products was determined. Assuming that the corrosion products are a mixture of $\mathrm{Fe}{\left(\mathrm{OH}\right)}_{2}$ and $\mathrm{Fe}{\left(\mathrm{OH}\right)}_{3}$ (the range of values is related to the adoption of different ratios of $\mathrm{Fe}{\left(\mathrm{OH}\right)}_{2}$ to $\mathrm{Fe}{\left(\mathrm{OH}\right)}_{3}$), then the mass of iron ions carried away to the solution is 35% to 45% of the total weight of iron ions in the corrosion.

Ultimately, the mass of iron ions calculated from Faraday’s law is in fact reduced due to the losses in the electrolysis process, and reduced by the mass of iron ions being part of the corrosion products carried away to the solution through the fracture and cracks in the resin shell. The said fact was taken into account in the research by introducing the ion mass reduction factor χ in the formulas describing computational electrochemical iron equivalent (9), (10) and (12):

(14) $$k_{\mathrm{comp}}={\chi k}_{\mathrm{eff}}=\chi \left(1-\lambda_{g-F}\right)k.$$

Since it was impossible to obtain precise data, the ion mass reduction factor χ was accepted as χ = 0.5, which was adopted in the numerical calculations. The calculations were also made for χ = 0.4 and χ = 0.35.

**Figure 7:**
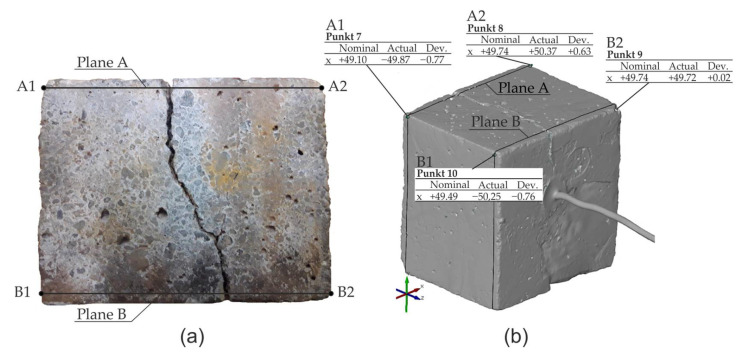
Measurements of crack opening during experimental tests: (**a**) schematic of the location of planes and control points, (**b**) reading of the displacement of points in the program GOM Inspect for the testing period t = 388 h.

The analysis of optical test results was limited to monitoring the displacement of the surface points of samples A1 and A2 in the plane A and points B1 and B2 in the opposite plane B (Figure 7a) in the abovementioned time periods. Figure 7b presents the reading of the displacement of the analyzed points in the program GOM Inspect (ZEISS, Oberkochen, Germany) for the test period t = 388 h. The relative elongation of the sample edges A1–A2 and B1–B2 can be identified as the arithmetic mean of the change of the width of crack openings on these edges of the sample. The measurement results as well as the results of calculations involving the elongation of the sides (widths of crack openings, presented in the further part of the work) at the abovementioned time intervals are presented in Figures 10 and 11.

## 4. Numerical Analysis

**Figure 8:**
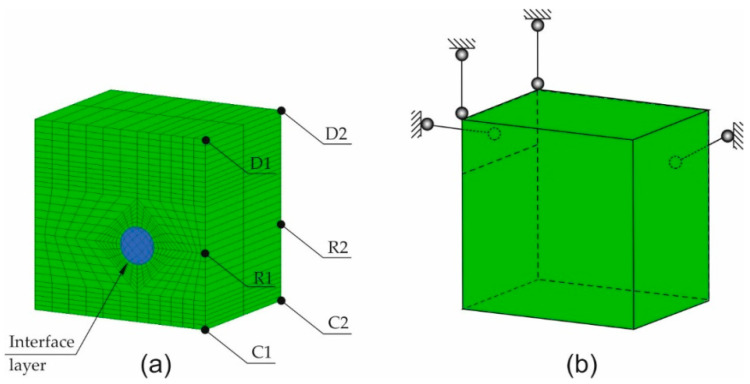
Static diagram of the sample adopted for numerical calculations: (**a**) finite element mesh, (**b**) boundary condition applied to the test sample–description in the text.

The FEM computer model of the tested samples was made using the software GiD-ATENA (GiD, Center for Numerical Methods in Engineering, Barcelona, Spain–Červenka Consulting, Praha, Czech Republic) and Matlab (MathWorks, Natick, United States of America). The model was made by the discretization of the sample into solid spatial finite elements with the interface layer between steel and concrete. The support method of the specimen and the finite elements mesh is presented in Figure 8a. The load that corresponded to subsequent increments in the volume of corrosion products was realized by applying the increments of strain tensor to the reinforcing bar, in the plane perpendicular to the axis of the reinforcing bar. The volumetric strain rates along the axis of the reinforcing bar that develop at the initial phase of the reinforcement corrosion process in line with the Equation (9) were ignored.

The sum of increments of the volumetric strain tensor produced by the accumulation of corrosion products in line with the Equations (9) and (10) was divided into 12 calculation steps in such a way that the increments of subsequent volumetric strains corresponded to the increments of successive time intervals between the measuring points. The computer simulation was carried out assuming that the average effective value of the electrochemical iron equivalent $k_{\mathrm{eff},\mathrm{avg}}=0.006271{\;\mu A}^{-1}{\mathrm{Year}}^{-1}$ and that the corrosion consisted entirely of iron hydroxide (II)-$\mathrm{Fe}{\left(\mathrm{OH}\right)}_{2}$ or iron hydroxide (III)-$\mathrm{Fe}{\left(\mathrm{OH}\right)}_{3}$. The parameters α, ϑ, dependent on the composition of the products, and the assumed thickness and porosity of the interface transition layer are presented in Table 4.

**Figure 9:**
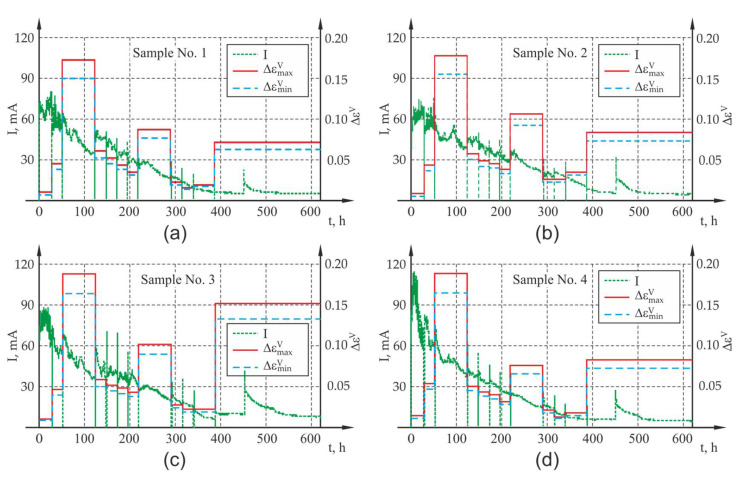
Coordinates of the tensors of effective increments of volumetric strains produced by corrosion products in reinforced concrete samples: (**a**) P1, (**b**) P2, (**c**) P3, (**d**) P4.

The increments of corrosion volumetric strains calculated with the dependencies (9) and (10) are compiled in the form of graphs in Figure 9a–d.

The critical time, the determination methodology of which was presented in Figure 3, was defined as the intersection point of two straight lines S1 and S2 approximated with the use of linear regression having the slopes $a_{i}$ and intercepts $b_{i}$. Calculations were made using the measured values of time and average values of the elongation of sample sides. Two values were employed for the calculations, one of which corresponded to the time interval tϵ (0,48〉 h for the straight line S1 and the other being the first 5 measured values from the time interval tϵ(48, 216〉 h for the straight line S2. It was assumed that the increments of the length and relative elongations of the sample edge had a linear character. The slopes of the straight line and the intercepts determined for the calculation data listed in Table 5 and Table 6 were, respectively: $a_{1}=0.00052$, $b_{1}=0.125$ for S1 and $a_{2}=0.00276$, $b_{2}=-0.1081$ for S2. The determined critical time, corresponding to the position of the abscissa of the intersection point of the straight lines S1 and S2, was $t_{\mathrm{cr}}=53.83$ h. The values adopted for the calculations are presented in Table 5 and Table 6.

The computer analysis of the concrete sample was performed using the ATENA system (Červenka Consulting, Praha, Czech Republic). The elastic-plastic-fracture material model with the interface steel–concrete connection, which makes use of the Rankin material model [27] in the field of tension, and the Menetrey–Willam model [27] in other cases was applied. The nonlinear geometry was turned on in the analysis. The material model defined in the system as Cc3DNonLinCementitious2 was applied. Strength parameters of the concrete were determined in line with the ATENA program manual for average cube strength under compression $f_{c,\mathrm{cube}}=65.89\;\mathrm{MPa}$. In addition, to improve the stability of the solution, the possibility of aggregate interlock and the shear factor were taken into account. The steel bar was defined by adopting elasto-plastic Huber-Misses-Hencky material [27]. The material parameters of steel and concrete (after the conversion from cube strength) and the interface layer used in the calculations are presented in Table 7, Table 8 and Table 9, respectively. The main role of the steel–concrete interface was to stabilize the results.

In order to assess the correlation of results obtained with the use of optical measurements and the computer model, a number of computer simulations were performed. The results obtained from the computer calculations were compared with the results of optical tests, and they are presented in Figure 8. As mentioned in point 3, in the calculations, allowance was made for a 50% share of corrosion products in relation to the value of corrosion strains obtained with the theoretical approach (χ= 0.5). For comparison, due to the discrepancy of results, χ=0.4 and χ=0.35 was also analyzed.

**Figure 10:**
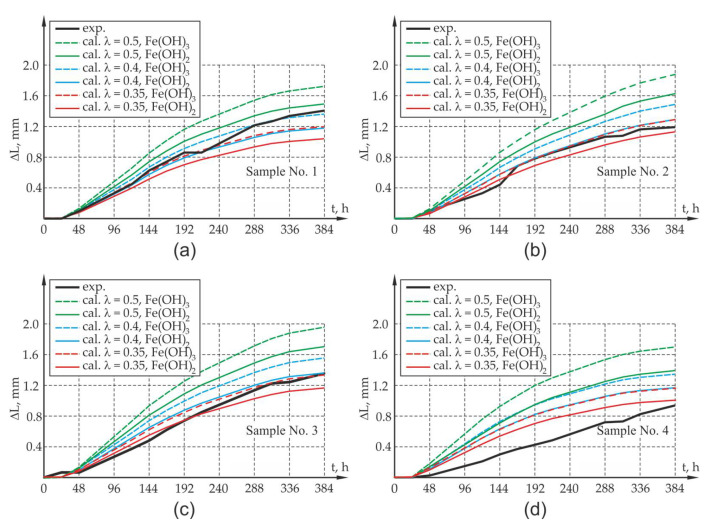
Average elongation values of edges ${\Delta L}_{A1A2}$ and ${\Delta L}_{B1B2}$ of the reinforced concrete sample together with calculation results for the 50%, 60% and 65% reductions of the impact intensity of corrosion products (λ = 0.5, λ = 0.4, λ = 0.35): (**a**) sample P1, (**b**) sample P2, (**c**) sample P3, (**d**) sample P4.

**Figure 11:**
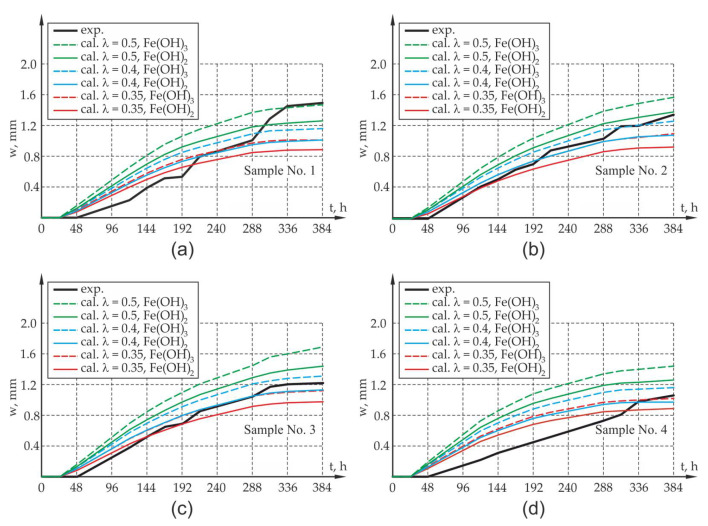
Average values of crack width at points of the elements’ edges on the sections A1A2 and B1B2 of the reinforced concrete sample at points R1 and R2 together with the calculation results for the 50%, 60% and 65% reductions of the impact intensity of corrosion products (λ = 0.5, λ = 0.4, λ = 0.35): (**a**) sample P1, (**b**) sample P2, (**c**) sample P3, (**d**) sample P4.

The assessment of the compliance of computer calculations with experimental tests involved the average value of the elongation of the wall edge perpendicular to the main direction of the sample crack (the calculated values of ${\Delta L}_{C1D1}$ and ${\Delta L}_{C2D2}$ specified in Figure 8 were compared with the measured values of ${\Delta L}_{A1A2}$ and ${\Delta L}_{B1B2}$—Figure 7) as well as the average calculated width of the crack at the edges C1D1 and C2D2 of the test element at Gauss points of the finite elements located in the vicinity of points R1 and R2 (Figure 8), which were compared with the measured value of average width of the crack at the edges A1A2 and B1B2. The results obtained from the computer simulations and from optical measurements are presented in Figure 10 and Figure 11.

When analyzing the results obtained from experimental research and computer simulations, agreement in respect of the quality of results and their quantitative estimation can be seen. The assessment of the compliance of research results with the results of theoretical calculations is complex due to large fluctuations in the research results which map the elongation of the sides and width of the crack of the sample and due to the small sample size from the viewpoint of statistical surveys. In the case of samples with an assumption of 50% reduction in impact intensity (χ = 0.5) caused by the flushing out of corrosion products, it can lead to the overestimation of the value offered by the mathematical model, which may be caused by the underestimation of the amount of corrosion products carried away by the solution. The results involving the elongation of the sides of the sample as well as the width of the crack opening in the test element differ from the results of experimental tests. Yet, the qualitative compliance in the assessment of the elongation and cracking of sample sides is noticeable both at the activation and local degradation stage of the propagation process of mechanical damage in the cover within the time range of 0–48 h as well as at further stages of the cracking process of the sample due to reinforcement corrosion.

Due to the experimental test result deviation in respect of the results of calculation for the 50% share of corrosion products in the degradation of the cover, an attempt was made in the next phase to assess the elongation of the sides of the elements and the width of crack openings in the sample in which 60% and 65% of the corrosion products were carried away to the electrolyte solution (40% and 35% of the load presented in Figure 9 is treated as the load of the concrete sample, χ= 0.4 and χ = 0.35). There is a rational premise that some proportion of the corrosion products at the measurement stage was washed out from the sample, as mentioned in Section 3 of the work. When analyzing the results of the experimental research and computer simulations obtained for test samples P1, P2, P3, agreement as to the quality of the results and their quantitative estimation can be observed. The results of experimental tests on the elongation of the sides of the sample, along with the width of the crack, are between the theoretically determined elongations for the assumed extreme compositions of corrosion products in the form of pure iron hydroxides II and III. There is a quantitative and qualitative compatibility of the elongations of the test element sides both at the activation and local degradation stage of the propagation process of mechanical damage in the cover (time (0,48〉 h) as well as at further cracking stages of the corrosion propagation process. The qualitative compliance of the calculations and experimental tests at the initial stage of the corrosion process is caused by the fact that the calculations allowed for the effects connected with the sealing up of free spaces and the propagation of micro-cracks in the transition layer, so-called CAR-cumulative accommodation region through the function β [13]. The discrepancies in the results of experimental tests and computer simulations are noticeable in relation to sample P4, although these discrepancies can be explained by more intense corrosion that occurred at the ends of the rebar (intense corrosion that developed under the polyester resin that protects the rebar face).

## 5. Conclusions

The paper presents a model that allows for the analysis of concrete cover cracking, taking into account the stages of the corrosion process, the time of tightening the corrosion products in the transition layer $t_{\mathrm{cr}}$, and the effective electrochemical equivalent of the reinforcing steel. The model can capture both the case of uniform and nonuniform corrosion on the circumference of the rebar. The obtained results of computer calculations were experimentally verified.

Summing up the obtained research results and the results of computer simulations, a number of conclusions involving the usefulness of the presented approach in assessing the opening width of cracks in reinforced concrete elements can be formulated.

### 5.1. Conclusion No. 1

The results obtained from numerical tests are qualitatively and quantitatively similar to the results of experimental research. This compliance can be observed for all stages characteristic of the process of reinforcing bar corrosion in concrete and involves the lack of mechanical impact at the initial stage of corrosion for the time $t\leq t_{0}$, the time interval until critical concentration time $t_{\mathrm{cr}}$ is reached, and the time interval in which full interaction is taking place between the corrosion products and the concrete of the cover $t\geq t_{\mathrm{cr}}$. Very important for the correctness of the obtained results is both the correct estimation of the critical time, which includes the condition of the cover tightening, as well as defining the effective value $k_{\mathrm{eff}}$ of the electrochemical equivalent of iron. Incorrect assumption of this parameter may lead in accordance with Equation (3) to large computational discrepancies, which was emphasized in Section 2 of this paper.

### 5.2. Conclusion No. 2

The compliance of the obtained test results is particularly important in the area of $t\epsilon \left(0,t_{\mathrm{cr}}\right)$. The fact that the function β was ignored in the calculation leads, in the case of theoretical calculations, to a rapid increment of displacements and propagation of damage in the element, which has a very negative impact on the accuracy of the obtained results and leads to qualitative and quantitative incompliance of the obtained computer simulation results. Similarly, the incorrect assessment of the critical time $t_{\mathrm{cr}},$ has a negative impact on the accuracy of the results.

### 5.3. Conclusion No. 3

The problem involving the washing out of corrosion products was adapted by way of estimation, but this did not affect the qualitative assessment of the obtained results. The quantitative results obtained with the 40% and 35% attempt at effective increments of corrosion strains are viewed as a satisfactory result.

## Figures and Tables

**Table 1:** Chemical composition of steel St3SX (S235JRG1) (%).

C	Si	Mn	P	S
0.18	0.30	0.32	0.04	0.04

**Table 2:** Chemical compositions of cement CEM I (%).

CaO	$SiO_{2}$	$Al_{2}O_{3}$	$Fe_{2}O_{3}$	$SO_{3}$	MgO	$Na_{2}O$	$K_{2}O$	$Cl^{-}$
64.5	19.3	5.97	2.71	2.29	0.82	0.37	0.36	0.049

**Table 3:** Comparison of the results of the gravimetric analysis and the calculations of the bar mass loss with the use of Faraday’s law for the samples complementing the first series of tests.

Test Specimen No.	Initial Mass $m_{0}$, (g)	Final Mass $m_{\tau }$ (g)	Bar Mass Loss $\bar{m}$, (g)	Bar Mass Loss ${\bar{m}}_{Fe^{2+}}$, (g)	Effective Electrochemical Equivalent of Iron $k_{eff},\;(g/\mu A\mathrm{Year})$
P5	239.115	228.485	10.630	16.529	0.005865
P6	236.183	222.040	14.143	18.959	0.006803
P7	240.176	229.675	10.501	15.582	0.006146
Avg.	238.491	226.733	11.758	17.023	0.006272

**Table 4:** Required parameters needed to determine the composition of corrosion products and the microstructure of the transition layer.

**Parameter**	**Value**
$\mathrm{Fe}{\left(\mathrm{OH}\right)}_{2}:$ parameter, α (1)	0.523
$\mathrm{Fe}{\left(\mathrm{OH}\right)}_{2}:$ parameter, ϑ (1)	2.09
$\mathrm{Fe}{\left(\mathrm{OH}\right)}_{3}:$ parameter, α (1)	0.622
$\mathrm{Fe}{\left(\mathrm{OH}\right)}_{3}:$ parameter, ϑ (1)	2.24
Porosity of transition layer, ε (1)	0.55
Width of transition layer, $w_{\mathrm{ws}}\times 10^{3}$(cm)	7.5

**Table 5:** Calculation parameters to determine the slope and the intercept in the simple regression S1.

Measurement Point i	1	2
Measurement time $t_{i}$, (h)	24	48
Average elongation of sample side ΔL, (mm)	0.025	0.038

**Table 6:** Calculation parameters to determine the slope and the intercept in the simple regression S2.

Measurement Point i	1	2	3	4	5
Measurement time $t_{i},$ (h)	125	144	168	192	216
Average elongation of sample side ΔL, (mm)	0.219	0.298	0.360	0.406	0.496

**Table 7:** Concrete material data for FEM calculations.

Material Property	Value
Modulus of elasticity E, (GPa)	38.28
Poisson’s ratio v, (1)	0.2
Medium tensile strength $f_{\mathrm{ctm}}$, (MPa)	3.99
Medium compressive strength $f_{\mathrm{cm}},$ (MPa)	56.4
Cracking energy $G_{F},$ (kN/m)	0.151
Maximum diameter of aggregate grains $d_{\max},$ (mm)	8
Active shear factor, (1)	20

**Table 8:** Interface layer material data for FEM calculations.

Material Property	Value
Stiffness in normal direction $k_{\mathrm{nn}}$, $\left(\mathrm{MN}/m^{3}\right)$	$2\times 10^{8}$
Stiffness in tangential direction $k_{\mathrm{tt}},\;\left(\mathrm{MN}/m^{3}\right)$	$2\times 10^{8}$
Interface tensile strength $f_{\mathrm{int}},\left(\mathrm{MPa}\right)$	0.3
Friction coefficient μ, (1)	0.1
Cohesion coefficient c,(MPa)	1

**Table 9:** Steel material data for FEM calculations.

Material Property	Value
Modulus of elasticity E, (GPa)	200
Poisson’s ratio v, (1)	0.3
Steel yield strength $f_{y},$ $\left(N/{\mathrm{mm}}^{2}\right)$	235
